# The Effect of Simulated In Vitro Digestion on Biological Activity of *Viburnum opulus* Fruit Juices

**DOI:** 10.3390/molecules26134086

**Published:** 2021-07-04

**Authors:** Nina Pietrzyk, Małgorzata Zakłos-Szyda, Małgorzata Redzynia, Anna Podsędek

**Affiliations:** Institute of Molecular and Industrial Biotechnology, Faculty of Biotechnology and Food Sciences, Lodz University of Technology, Stefanowskiego 2/22, 90-537 Łódź, Poland; nina.pietrzyk@dokt.p.lodz.pl (N.P.); malgorzata.zaklos-szyda@p.lodz.pl (M.Z.-S.); malgorzata.redzynia@p.lodz.pl (M.R.)

**Keywords:** *V. opulus*, phenolic compounds, in vitro digestion, cell cultures, lipid accumulation, glucose uptake, insulin secretion

## Abstract

In the present study, an in vitro digestion method has been used to assay the influence of the physiological conditions in the mouth, stomach, and intestine on the stability and activity in different cell models of the main phenolic compounds from *Viburnum opulus* fresh juice (FJ), phenolic-rich juice (PJ), and the bioavailable fractions (DFJ and DPJ). The data obtained indicate that the *V. opulus* samples achieved after in vitro digestion had an influence on cellular glucose and lipid metabolism. The bioavailable fraction of both digested juices stimulated glucose uptake and decreased lipid accumulation by L6 myoblasts and HepG2 hepatocytes. Both DFJ and DPJ reduced the secretion of inflammatory cytokines by 3T3-L1 adipocytes: interleukin-6 (IL-6) and tumor necrosis factor-α (TNF-α). Simultaneously, DFJ and DPJ enhanced oxidative stress in MIN6 cells and decreased glucose-stimulated insulin secretion (GSIS). UPLC–MS analysis revealed qualitative and quantitative changes in hydroxycinnamic acids. In particular, the content of chlorogenic acid decreased drastically; its content in the bioavailable fraction was almost 7 times and 30 times lower than in the FJ and PJ, respectively. Our results suggested that although the phenolic compounds of *V. opulus* juices undergo transformation during digestion, they are still potent antioxidant agents with biological activity.

## 1. Introduction

*Viburnum opulus* L. belongs to the *Viburnum* L. genus from the *Adoxaceae* family and this shrub is common in natural habitats in Europe, North Asia, and North Africa, and also in the central zone of Russia. The fruit of *V. opulus* has a light red, red, or dark red skin, is bitter with a strong astringent taste, and is therefore not preferred for direct consumption [1]. However, these fruits are used for preparing jam, jelly, cordials, and liqueurs, and “Kalinnikov” pies as well as herbal teas [2,3]. In Turkey, they are used for the production of a traditional non-alcoholic fermented beverage called “Gilaburu juice” [4]. The *V. opulus* fruits and fruit juice are widely used for medicinal purposes as a useful remedy against kidney and stomach problems, high blood pressure, cough and cold, tuberculosis, rheumatic aches, liver disease, and diabetes [1]. The published results of in vitro studies indicate antimicrobial [5,6,7], antidiabetic [8], anti-obesity [9,10], and antioxidant [2,11,12,13,14] activities. Furthermore, the health-promoting properties of *V. opulus* fruit and fruit juice have also been demonstrated in studies using various cell lines. For example, our previous study showed that *V. opulus* fruit components decreased the uptake of fluorescent glucose analogue 2-(N-(7-nitrobenz-2-oxa-1,3-diazol-4-yl)amino)-2-deoxyglucose by human adenocarcinoma Caco-2 cells [15]. The next study demonstrated that *V. opulus* fruit juice and juice enriched with phenolic compounds decreased glucose-stimulated insulin secretion, increased insulin secretion at a low glucose concentration, and intensified free fatty acid uptake and lipid accumulation in the mouse insulinoma cell line MIN6 [16]. Additionally, *V. opulus* fruit phytocompounds influenced the accumulation of lipids and expression of lipogenic proteins involved in metabolic disorders in HepG2 cells [17]. It has been also observed that *V. opulus* fruit juice decreased levels of intracellular reactive oxygen species (ROS) in mouse pancreatic beta MIN6 cells, as well as in mouse differentiated adipocytes 3T3-L1 and in human hepatoma HepG2 cells [9,10,16,17]. Karakurt et al. showed that methanol extract of *V. opulus* fruit inhibits the proliferation of human colon cancer cells DLD-1 and HT-29 by increasing DLD-1 cell apoptosis and cell cycle arrest at the G2 phase in HT-29 cells [18].

The demonstrated results of in vitro studies show the promising health benefits of *V. opulus* fruit. However, the physiological importance of an orally administered phytocompound depends on its availability for intestinal absorption and subsequent interaction with target tissues. During the digestion process, food components are transformed as a consequence of a variety of digestive enzymes, variance in the pH values, and other physical and biochemical aspects. However, the bioavailability of the active compounds is generally neglected in the vast majority of the in vitro studies. Our previous study identified *V. opulus* fruit juice as a rich source of phenolic compounds, with the highest content of chlorogenic acid, followed by flavanols and anthocyanins [10]. To the best of our knowledge, only one report concerns the effect of the in-vitro-simulated digestion process on phenolic compounds’ stability and the antioxidant activity of methanolic and aqueous *V. opulus* fruit extracts [19]. The results showed that phenolic compounds were negatively influenced by in vitro simulation of human digestion because total phenolics decreased by 63.23% and 40.60% for the methanolic and aqueous extract, respectively. The potential bioavailability index (ratio of amounts of phenolics in serum available fraction to the phenolic content in undigested sample) was estimated as 36.77–59.40% for total phenolics and 38.90–56.40% for chlorogenic acid (a major metabolite of *V. opulus* fruit), depending on the type of extract. Moreover, the cited studies showed lower free radical scavenging capacity and metal reducing capacity of serum available and colon available fractions than undigested extract.

Therefore, the use of plant extracts as well as isolated bioactive compounds previously subjected to digestion in cell-based research is justified. Thus far, some studies have confirmed the effect of bioactive phytocompounds after the in vitro digestion process on their biological activity determined in cell models. Stanisavljević et al. showed that chokeberry juice digested in the presence of the food matrix markedly reduced the proliferative rate of Caco-2 cells [20]. Similarly, the digested extract of cooked purple potatoes retained the ability to reduce Caco-2 cell proliferation, although to a lesser extent compared to the extract without digestion [21]. In contrast, Gutiérrez-Grijalva et al. observed an increase in cellular antioxidant activity in Caco-2 cells of oregano polyphenols after gastrointestinal digestion [22]. Thus, the aim of this study was to determine the changes in hydroxycinnamic acid content as the main phenolic component of *V. opulus* juice during in-vitro-simulated mouth and gastrointestinal digestion of fresh juice (DFJ) and phenolic-rich juice (DPJ). As a cellular model for biological study, different types of cells were chosen, whose activity may be influenced by the bioavailable fraction of extracts obtained after their digestion and absorption, i.e., myoblasts (L6 cell line), hepatocytes (HepG2 cell line), insulinoma β cells (MIN6 cell line), and adipocytes (3T3-L1 cell line). These cellular models are commonly used in studies exploring the regulation of lipids and carbohydrate metabolism. Therefore, the research especially focused on the influence of digested and absorbed fractions of *V. opulus* preparations on cellular glucose uptake and lipid metabolism by L6 and HepG2 cells, the adipogenesis and secretion of pro-inflammatory cytokines by 3T3-L1 cells, as well as insulin secretion by MIN6 cells. To the best of our knowledge, this is the first study demonstrating the cell-based in vitro activity of *V. opulus* juice after digestion treatment, which additionally compares the effect of the digestion process on selected cellular effects.

## 2. Results

### 2.1. The Influence of In-Vitro-Digested V. opulus Juice Samples on Metabolic Activity of Cells

**Figure 1:**
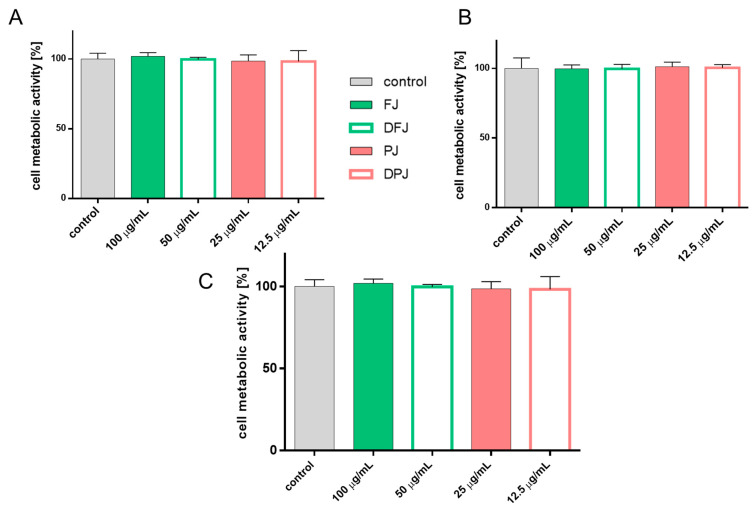
The influence of *V. opulus* samples on HepG2 (**A**), L6 (**B**), and Caco-2 (**C**) cells’ metabolic activity after 24 h exposure of fresh juice (FJ), purified juice (PJ), and bioavailable fractions after digestion process (DFJ, DPJ) at non-cytotoxic concentration (IC_0_); control cells were not exposed to any compound except vehicle; values are means ± standard deviations from at least five independent experiments, *n* ≥ 9; statistical significance was calculated versus control cells (untreated).

Previous studies revealing the biological potential of *V. opulus* preparations on metabolic activity allowed the determination of the maximum concentration without cytotoxic effect (IC_0_) on the metabolic activity of various types of cell lines, i.e., MIN6, 3T3-L1, and HepG2 [10,15,16,17]. Based on the previous results, the fresh juice (FJ) at a concentration of 100 µg of dry weight of juice/mL, and purified juice (PJ) at 25 µg/mL, were used as the reference values to compare the biological effects of the corresponding digested preparations. The influence of bioavailable fractions that had passed through the semipermeable membrane during in vitro intestinal digestion of *V. opulus* for both juices was estimated at a concentration of 50 µg/mL for digested fresh juice (DFJ) and 12.5 µg/mL for digested purified juice (DPJ). The use of two-times-lower doses for digested samples resulted from the purification process of DFJ and DPJ, which caused a two-times reduction in their volume compared to undigested samples. As presented in Figure 1A,B, all of the tested *V. opulus* samples had no effect on the cell metabolic activity of HepG2 and L6 cells. Because the digested food reached the intestinal epithelium as the first target organ, the effect of samples on Caco-2 cells’ metabolic activity was additionally studied (Figure 1C).

### 2.2. The Effect of Digested V. opulus Juice Samples on Biological Activity of HepG2 Cells

Reactive oxygen species (ROS) locally produced in the hepatic tissue seems to be involved in the pathogenesis of metabolic disorders [23]. It is well known that chronic free fatty acid (FFA) elevation leads to steatosis of hepatocytes and is connected to excessive oxidative stress [24]. Therefore, the influence of phenolic compounds obtained from digested bioavailable fractions of *V. opulus* juices (DFJ and DPJ) on the cellular antioxidant activity using a DCF probe and cellular FFA uptake (TF2-C12 analogue) was determined.

**Figure 2:**
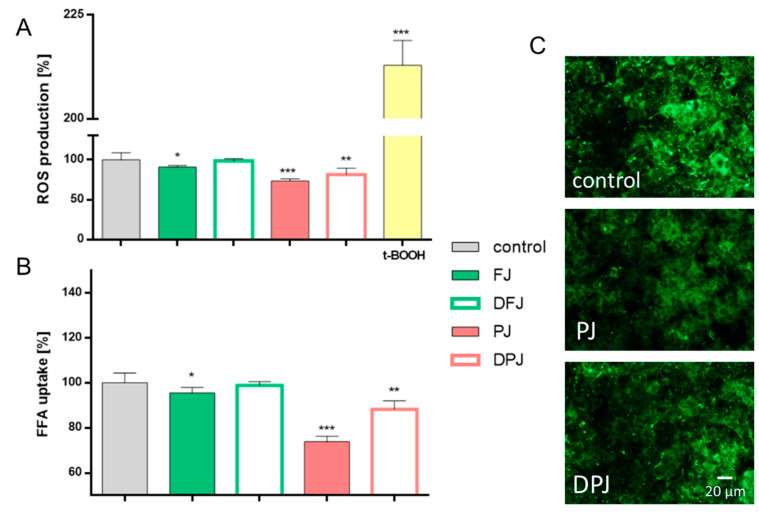
The effect of *V. opulus* juice samples on intracellular ROS generation (**A**) and on fatty acid analogue TF2-C12 uptake (**B**) in HepG2 cells after 24 h incubation with fresh juice (FJ), digested fresh juice (DFJ), purified juice (PJ), and digested purified juice (DPJ); for positive control for ROS generation, 500 µM *tert*-BOOH was used; values are means ± standard deviations from at least five independent experiments, *n* ≥ 9; statistical significance was calculated versus control cells (untreated), * *p* ≤ 0.05, ** *p* ≤ 0.01, *** *p* ≤ 0.001. Cellular uptake of TF2-C12 analogue visualized under a fluorescent microscope (200× magnification) (**C**).

Hepatoma cells’ pre-incubation with FJ and PJ at IC_0_ decreased the intracellular ROS level by 10% for FJ and 25% for PJ compared to the control cells treated with the vehicle only (Figure 2A). The digestive process resulted in the loss of antioxidant activity by DFJ. On the other hand, DPJ lowered the cellular oxidative stress by nearly 10%. Nevertheless, PJ decreased the intracellular ROS level by almost 25% and it was the most effective antioxidant.

The lower activity of *V. opulus* juice samples obtained after digestion was also observed in a further study. As shown in Figure 2B, the level of fluorescent free fatty acid analogue TF2-C12 incorporated in the presence of PJ was decreased by 25%, whereas DPJ lowered fluorescence by 12%. Although HepG2 cells’ treatment with FJ reduced FFA uptake by 5%, DFJ had no effect in this regard.

**Figure 3:**
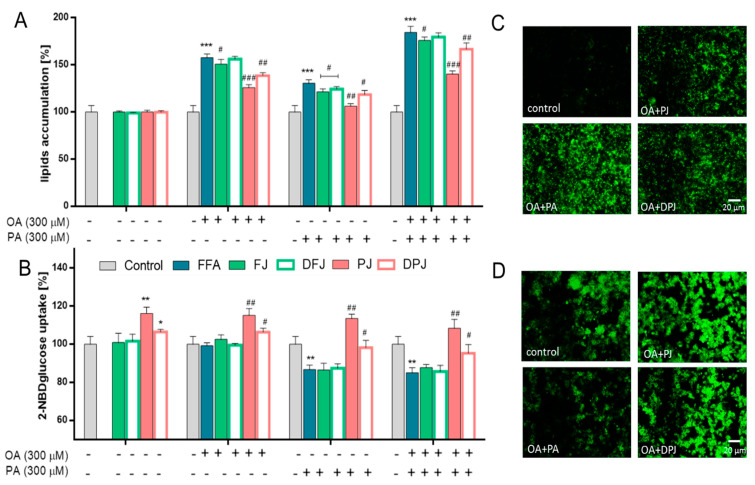
The influence of *V. opulus* fresh juice (FJ), digested fresh juice (DFJ), purified juice (PJ), and digested purified juice (DPJ) on the accumulation of lipid droplets (**A**) and the effect on fluorescent glucose analog 2-NBDG uptake (**B**) in HepG2 cells after 24 h co-incubation with 300 µM oleic acid and/or palmitic acid; control cells were not exposed to any compound except vehicle; values are means ± standard deviations from at least five independent experiments, *n* ≥ 9; statistical significance was calculated versus control cells (untreated), * *p* ≤ 0.05, ** *p* ≤ 0.01, *** *p* ≤ 0.001 and versus positive cells (treated with fatty acids), # *p* ≤ 0.05, ## *p* ≤ 0.01, ### *p* ≤ 0.001. Cells visualized under fluorescent microscope at 200× magnification with FITC filter after Nile Red staining (**C**) and after 2-NBDG uptake (**D**).

To evaluate the cytoprotective potential of digested *V. opulus* juice samples against steatosis, HepG2 cells were co-incubated with IC_0_ concentration of samples in the presence of a non-cytotoxic concentration (300 µM) of oleic acid (OA) or/and palmitic acid (PA) for 24 h. Due to the fact that high levels of free fatty acids, especially palmitic acid, under steatosis conditions lead to insulin resistance and glucose uptake disturbance [25,26], in this research, glucose analogue uptake (2-NBDG) was also evaluated (Figure 3).

As presented in Figure 3A, fatty acid treatment led to a significant increase in the intracellular formation of lipid droplets, and PA and OA + PA treatment resulted in decreased uptake of glucose analogue (Figure 3B). In this study, HepG2 cells were treated with digested purified juice (DPJ) and simultaneously with purified juice (PJ) without digestion treatment, which allowed a direct comparison of the digestion effect on the biological activity. Compared to PJ, the DPJ decreased its inhibitory potential on lipid accumulation by 10–15% (even 25% for OA + PA). The simultaneously observed decrease in lipid accumulation in PJ-treated cells was in agreement with previously published data by the authors (lipid accumulation decrease by 24–44% in comparison to FFA-treated cells) [17]. These data were confirmed by microscopic observations—in Figure 3C, it can be observed that there was a reduction in the number of lipid droplets formed in FFA-treated cells after PJ and DPJ co-incubation. Our previously published research showed that HepG2 cells’ co-incubation with PJ led to a significant decrease in lipid accumulation (by 30%, 25%, and 45% for OA, PA, and OA + PA, respectively) and enhanced 2-NBDglucose uptake (by 16% for incubation itself and for OA co-incubation, 25% for PA and OA + PA, respectively) [17]. Additionally, the fluorescent signal for 2-NBDG uptake was enhanced in cells after PJ and DPJ treatment in the presence of OA + PA (Figure 3D).

### 2.3. The Effect of Digested V. opulus Juice Samples on Biological Activity of L6 Cells

**Figure 4:**
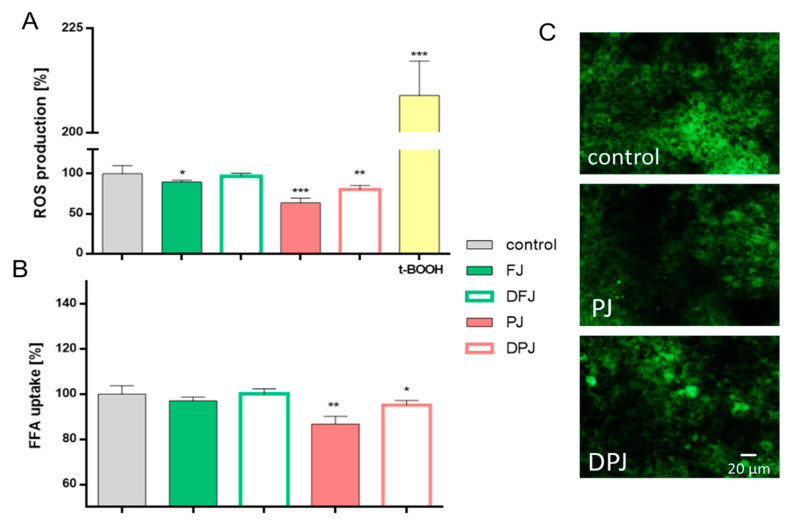
The effect of *V. opulus* juice samples on intracellular ROS generation (**A**) and the effect on fatty acid analogue TF2-C12 uptake (**B**) in L6 cells after incubation with fresh juice (FJ), digested fresh juice (DFJ), purified juice (PJ), and digested purified juice (DPJ) after 24 h incubation; for positive control for ROS generation, 500 µM *tert*-BOOH was used; values are means ± standard deviations from at least five independent experiments, *n* ≥ 9; statistical significance was calculated versus control cells (untreated), * *p* ≤ 0.05, ** *p* ≤ 0.01, *** *p* ≤ 0.001. Cellular uptake of TF2-C12 analogue visualized under a fluorescent microscope (200× magnification) (**C**).

Enhanced lipolysis resulting from adipocyte dysfunction causes muscle lipid accumulation under the condition of insulin resistance. Therefore, in this study, we decided to evaluate the biological effect of undigested and digested *V. opulus* juice samples on rat skeletal muscle L6 cells (Figure 4).

Conducted studies using the fluorescent DCF probe showed that FJ decreased the intracellular ROS level by almost 10% (Figure 4A), which is comparable to activity previously observed [10,15,16,17]. The purification process of *V. opulus* juice resulted in a significant increase in cellular antioxidant activity in L6 cells, where ROS production was decreased by 37% compared to control cells. Moreover, this activity was still noticeable after the digestion process—DPJ treatment decreased the intracellular ROS level by 20%. Comparable potential was also observed for FFA analogue uptake (Figure 4B), where PJ and DPJ decreased the fluorescent signal by 15% and 5%, respectively. Microscopic observation confirmed the obtained data (Figure 4C).

**Figure 5:**
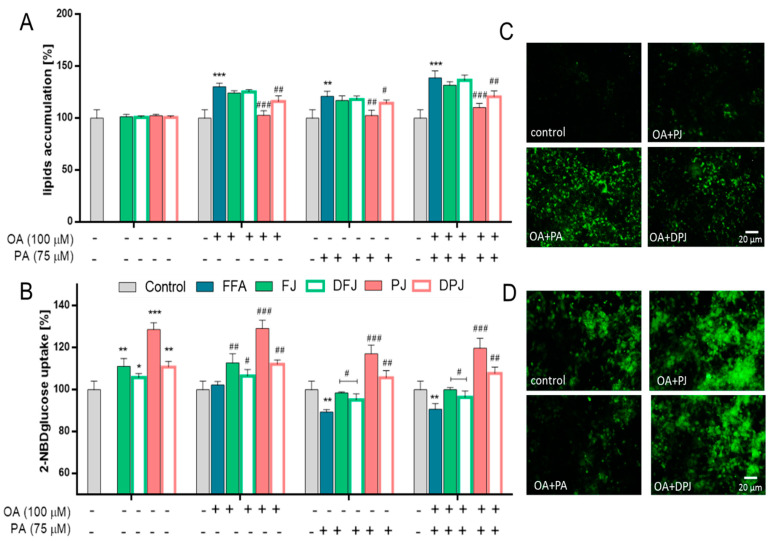
The influence of *V. opulus* fresh juice (FJ), digested fresh juice (DFJ), purified juice (PJ), and digested purified juice (DPJ) on the accumulation of lipid droplets (**A**) and the effect on fluorescent glucose analog 2-NBDG uptake (**B**) in L6 cells after 24 h co-incubation of cells with oleic acid (100 μM) and/or palmitic acid (75 μM); control cells were not exposed to any compound except vehicle; values are means ± standard deviations from at least five independent experiments, *n* ≥ 9; statistical significance was calculated versus control cells (untreated), * *p* ≤ 0.05, ** *p* ≤ 0.01, *** *p* ≤ 0.001 and versus positive cells (treated with fatty acids), # *p* ≤ 0.05, ## *p* ≤ 0.01, ### *p* ≤ 0.001. Cells visualized under fluorescent microscope at 200× magnification with FITC filter after Nile Red staining (**C**) and after 2-NBDG uptake (**D**).

Figure 5A presents results proving that *V. opulus* PJ has significant activity in decreasing lipid accumulation in FFA-induced steatosis conditions. Treatment of L6 cells with PJ decreased lipid accumulation by 30%, 20%, and 28% for OA, PA, and OA + PA co-incubation, respectively. DPJ also lowered the rate of lipid accumulation by 15%, 6%, and 18% (compared to FFA-treated cells as a positive control). In Figure 5C, it can be observed that there was lowered fluorescence generated from lipid droplets after cells’ PJ and DPJ treatment in the case of OA + PA co-incubation. The drastic elevation of free fatty acids during steatosis of cells is accompanied by a strong reduction in glucose uptake in skeletal muscle cells [27]. Therefore, the effect of *V. opulus* juice samples on 2-NBDglucose analogue uptake in L6 under increased levels of OA and/or PA was evaluated. All of the tested *V. opulus* juice samples showed an ability to increase 2-NBDG uptake (Figure 5B). The strongest enhancing effect revealed PJ, which elevated 2-NBDG uptake by nearly 30% in comparison to untreated cells. The FJ and DPJ increased fluorescence by 10%; therefore, the effect was lower than for PJ. It is worth noting that palmitic acid treatment (PA and OA + PA) resulted in a decrease in 2-NBDglucose analogue uptake by 10% compared to control cells. This effect was unnoticeable for cells treated with OA despite its higher influence on lipid accumulation than PA. This result confirmed that saturated palmitic acid is a higher insulin resistance inducer than unsaturated fatty acids [28,29], which also is in agreement with previously published results for HepG2 cells [17]. It needs to be emphasized that tested *V. opulus* juice samples enhanced 2-NBDglucose analogue uptake in cells treated with PA and OA+PA by 10–30%, which was also confirmed by microscopic observation (Figure 5C). Potential to enhance glucose analogue uptake by L6 cells was also observed after treatment with DFJ (by 10% for OA + PA).

### 2.4. The Effect of Digested V. opulus Juice Samples on Insulin Secretion of MIN6 Cells

**Figure 6:**
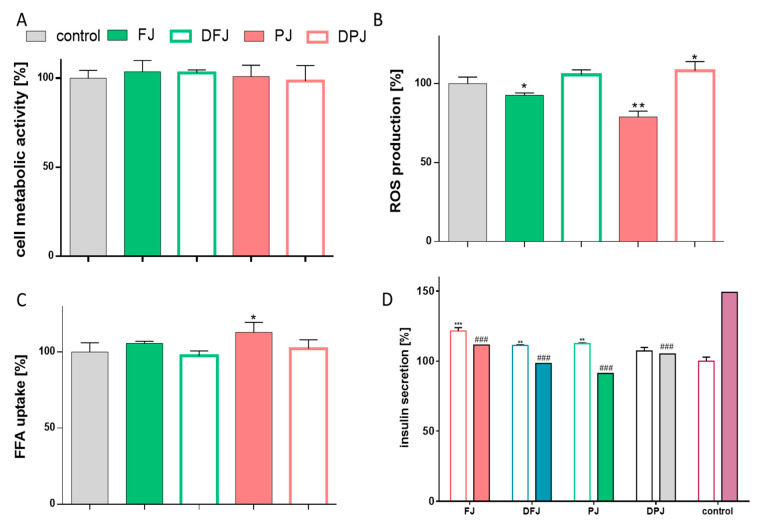
The influence of *V. opulus* samples on MIN6 cells’ metabolic activity after 24 h exposure of fresh juice (FJ), digested fresh juice (DFJ), purified juice (PJ), and digested purified juice (DPJ) at non-cytotoxic concentration (IC_0_) (**A**); intracellular ROS generation (**B**); fatty acid analogue TF2-C12 uptake (**C**); insulin secretion by cells in low (open bars) or high (closed bars) glucose conditions (**D**). Control cells were not exposed to any compound except vehicle; values are means ± standard deviations from at least three independent experiments, *n* ≥ 9; statistical significance was calculated versus control cells (untreated), for low glucose (*) or high glucose (#); * *p* ≤ 0.05, ** *p* ≤ 0.01, *** *p* ≤ 0.001; ; # *p* ≤ 0.05, ## *p* ≤ 0.01, ### *p* ≤ 0.001.

Taking into account that the pancreas is involved in glucose and lipid metabolism regulation, the influence of digested *V. opulus* juice samples on the mouse insulinoma MIN6 line was determined. As presented in Figure 6A,B, *V. opulus* FJ at a noncytotoxic concentration decreased intracellular oxidative stress in MIN6 cells. Purified juice (PJ) was the most effective and reduced the ROS level by almost 25% in comparison to the control cells. Surprisingly, the digestion process deprived the preparation of the antioxidant activity in the studied cellular model. Whereas DFJ had no effect on the ROS level, the DPJ increased oxidative stress by at least 5%. Additionally, it was confirmed that PJ stimulated free fatty acid uptake by 7%, which can further lead to increased lipid accumulation and metabolism deregulation in β-cells (Figure 6C). In this regard, the digestion process revealed a protective effect on DPJ potential—after MIN6 cells’ incubation with the FFA, the fluorescent analogue accumulation level was comparable to the control cells.

The MIN6 cell line displays characteristics of pancreatic β-cells insulin secretion in response to glucose [16]. Therefore, the effect of *V. opulus* undigested and digested juices on glucose stimulated insulin secretion (GSIS) was determined. As presented in Figure 6D, the exposure of MIN6 to 20 mM glucose enhanced insulin secretion by almost 50%. All studied *V. opulus* samples had an inhibitory effect on GSIS, reducing the insulin level by 35–60% in comparison to high-glucose-treated cells. PJ was a stronger reducer of GSIS than FJ. On the other hand, DPJ had a 10% lower negative effect on insulin secretion than PJ. At a low-glucose concentration (2 mM), all samples showed increased insulin secretion by 10–20% and FJ was the strongest activator of this process.

### 2.5. The Effect of Digested V. opulus Juice Samples on Adipogenesis of 3T3-L1 Cells

**Figure 7:**
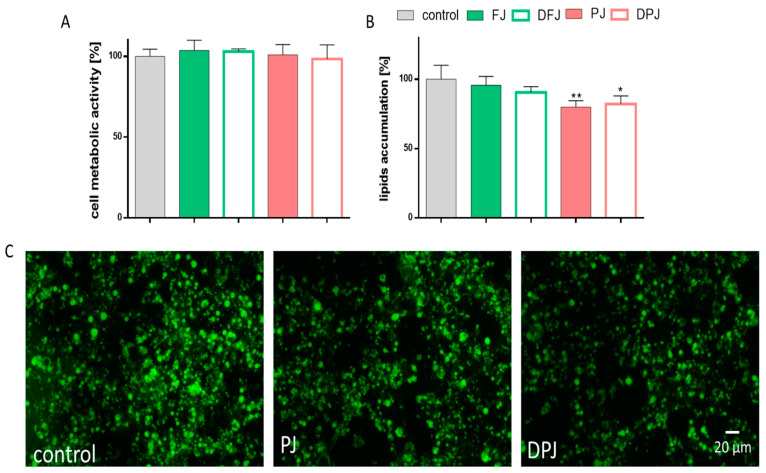
The influence of *V. opulus* fresh juice (FJ), digested fresh juice (DFJ), purified juice (PJ), and digested purified juice (DPJ) at non-cytotoxic concentration (IC_0_) on differentiated 3T3-L1 cells’ metabolic activity after 7-day incubation (**A**); the accumulation of lipid droplets (**B**); control cells were not exposed to any compound except vehicle; values are means ± standard deviations from at least three independent experiments, *n* ≥ 9; statistical significance was calculated versus control cells (untreated), * *p* ≤ 0.05, ** *p* ≤ 0.01. Cells visualized under fluorescent microscope at 200× magnification with FITC filter after Nile red staining (**C**).

Because adipocytes play a role in energy homeostasis and insulin sensitivity via adipogenesis and secretion of adipocytokines, next, the influence of digested *V. opulus* juice samples was studied on the mouse 3T3-L1 preadipocytes. The adipogenesis process is connected to the differentiation of preadipocytes to mature adipocytes. As a result, the formation of lipid droplets within the cells occurs. As presented in Figure 7A,B, the PJ and DPJ at IC_0_ significantly reduced the accumulation of lipid droplets compared to the control cells by at least 20%. These data were confirmed by microscopic observation of cells with fluorescence dye that accumulates in lipid droplets **(**Figure 7C). Both FJ and DFJ also decreased the fluorescence level in adipocytes; however, the observed effect did not exceed 10%.

**Figure 8:**
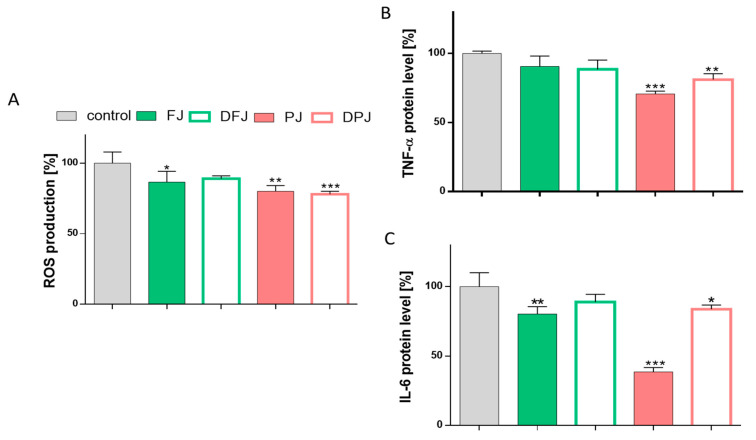
The influence of *V. opulus* fresh juice (FJ), digested fresh juice (DFJ), purified juice (PJ), and digested purified juice (DPJ) at non-cytotoxic concentration (IC_0_) on intracellular ROS generation in differentiated 3T3-L1 cells after 7-day incubation (**A**); the secretion of tumor necrosis factor α (TNF-α) (**B**); the secretion of interleukin-6 (IL-6) (**C**); control cells were not exposed to any compound except vehicle; values are means ± standard deviations from at least three independent experiments, *n* ≥ 9; statistical significance was calculated versus control cells (untreated), * *p* ≤ 0.05, ** *p* ≤ 0.01, *** *p* ≤ 0.001.

The enlargement of adipocytes generates an elevation in ROS production and stimulates the secretion of inflammatory cytokines related to obesity and insulin resistance. Figure 8A shows that all *V. opulus* juice samples reduced intracellular ROS production in differentiated adipocytes. Undigested PJ decreased oxidative stress by 20%, while FJ by 15%. Whereas FJ after the digestion process (DFJ) had potential lower by 5% than the undigested sample, the DPJ had a comparable effect to PJ. The same samples reduced the secretion of TNF-α protein to 75–80% (Figure 8B), while no relevant effect was observed for FJ and DFJ. In contrast, all studied *V. opulus* juice samples downregulated the secretion of IL-6 protein to 40–70%. The most intensive reduction was observed for PJ (by 60%) and FJ (by 20%), whereas the digested samples decreased the IL-6 level to 10–15% in comparable way (Figure 8C).

### 2.6. Hydroxycinnamic Profiles of V. opulus Fresh (FJ) and Purified (PJ) Juice before and after In Vitro Digestion

**Figure 9:**
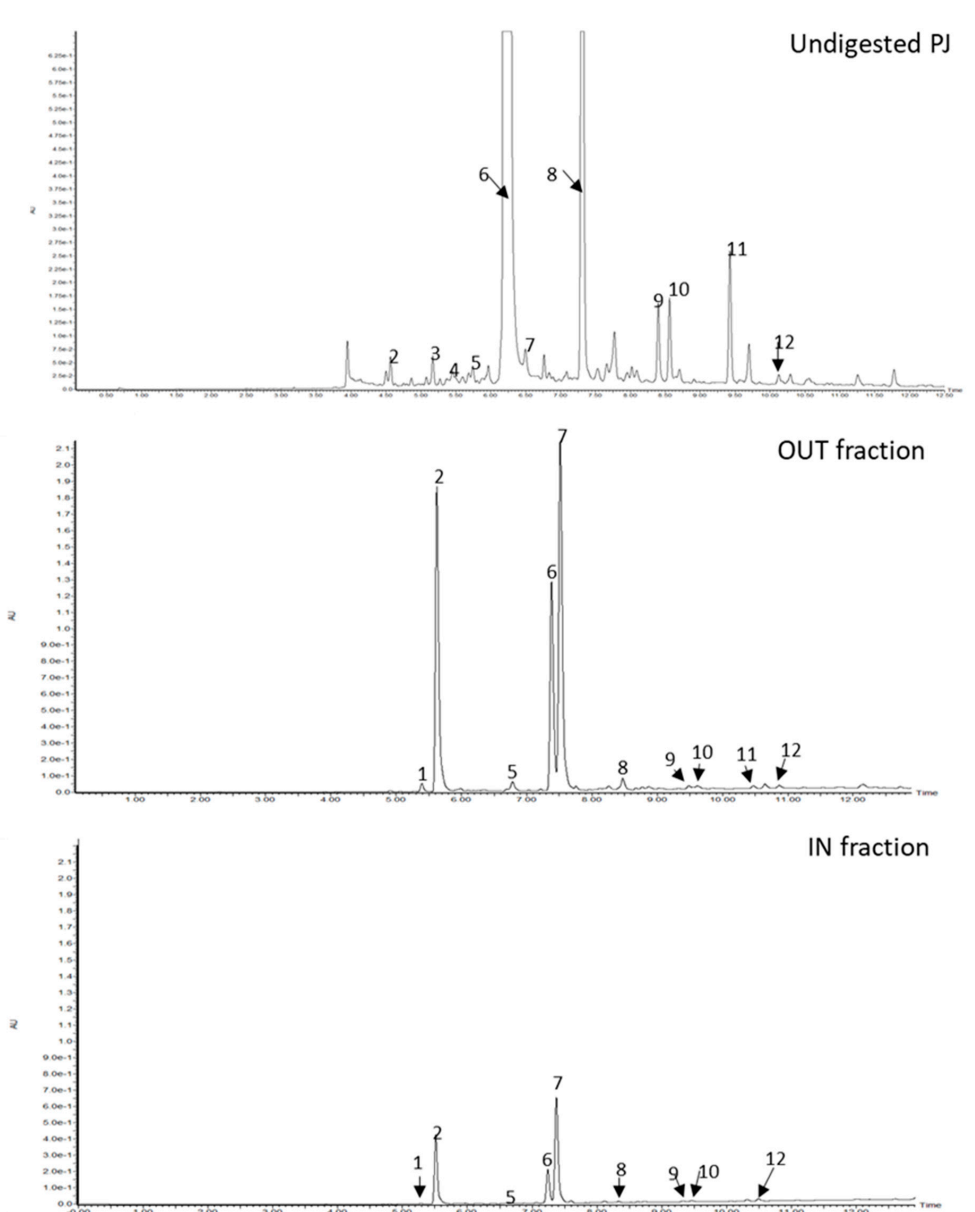
Chromatograms of undigested purified juice (PJ) of *V. opulus* fruit and after in vitro digestion process (OUT and IN fractions) registered at 320 nm. Refer to Table 1 for the identification of each numbered peak.

Both the *V. opulus* juice samples were subjected to an in vitro digestion procedure designed to simulate in vivo digestion. The contents of individual hydroxycinnamic acids in both undigested and digested juices are shown in Figure 9 and Table 1. The main component of both undigested juices was chlorogenic acid (peak 6). It constituted 89.96% and 91.31% of the total amounts of hydroxycinnamic acids in FJ and PJ, respectively. As a result of the three-stage digestion process, its content significantly decreased by 77.17% and as much as 96.14% in the case of FJ and PJ, respectively. It should be noted that the total content of hydroxycinnamic acids in DFJ was 31.65% lower than in the undigestible sample. For comparison, the total content of this group of phenolic acids after PJ digestion decreased by 87.60%. The loss of chlorogenic acid content resulted in a significant increase in the content of its isomers, such as neochlorogenic acid (peak 2) and cryptochlorogenic acid (peak 7). For example, the concentration of neochlorogenic acid increased from 0.007 to 1.444 mg/g and cryptochlorogenic acid from 0.004 to 1.122 mg/g due to digestion of FJ. In the case of PJ, the changes in content were from 0.215 to 24.024 mg/g and from 0.484 to 27.493 mg/g for neochlorogenic and cryptochlorogenic acid, respectively. Moreover, the digestion process of FJ and also PJ resulted in complete degradation of the three and two caffeoylquinic acid derivatives, respectively. On the other hand, the appearance of a new derivative with the negative molecular ion [M − H]^−^ equal to 353 *m*/*z* (peak 1) in DFJ was noted. The OUT fraction, representing bioavailable hydroxycinnamic acids, was characterized by a higher content of all identified compounds than the IN fraction (colon available). Their total content in the OUT fraction was 62.14% higher than in the IN fraction in the case of FJ and 4.5 times higher for the PJ.

## 3. Discussion

There are many scientific reports indicating the positive health influence of phenolic compounds isolated from selected edible or nonedible plants [30,31]. This potential is attributed to phenolics’ high antioxidant activity, the ability to restrain steatosis and the expression of lipogenic proteins, as well as improvement of glucose uptake and reduction of insulin resistance, among others [17,32,33,34,35]. On the other hand, there are few reports of the above-mentioned in vitro activities observed for plant preparations rich in phenolic compounds after the digestion process. In the presented study, the influence of digestion of *V. opulus* juice samples on biological activity was evaluated. The direct comparison was performed between fresh juice (FJ) and purified juice (PJ) and their corresponding bioavailable fractions (DFJ and DPJ) obtained after tree-step static in vitro digestion. The phenolic composition of FJ and PJ described in the previous article included hydroxycinnamic acids, flavanols, anthocyanins, and flavonols [10]. The percentage of these groups in the total content of phenolic compounds in FJ was 77.50%, 19.52%, 2.62%, and 0.37%, respectively. For comparison, hydroxycinnamic acids accounted for 80.46%, flavanols 16.30%, anthocyanins 2.92%, and flavonols 0.37% of the total phenolics in PJ. The current results show the significant influence of the digestion process on the transformation of hydroxycinnamic acids—the dominant phenolic components of the tested samples obtained from *V. opulus* fresh fruits (Table 1). The total contents of hydroxycinnamic acids in DFJ and DPJ were 31.65% and 87.60% lower, respectively, than in the undigestible sample. Moreover, chlorogenic acid content significantly decreased by 77.17% and as much as 96.14% during digestion of FJ and PJ, respectively. On the other hand, the loss of chlorogenic acid content resulted in a significant increase in the content of neochlorogenic acid and cryptochlorogenic acid. Other authors have also suggested the biotransformation of parental phenolic compounds in undigested plant material during the digestive process in the gastrointestinal tract, mainly due to changes in pH at each digestive phase and, to a lesser extent, by the action of digestive enzymes [22,36,37]. Moreover, the lower losses of hydroxycinnamic acids in the case of FJ digestion compared to PJ indicate the protective effect of other FJ components (removed during juice purification by SPE) towards phenolic compounds. The influence of the composition of the food matrix on the stability of phenolic compounds during simulated digestion was also shown by other studies [20,38,39]. An application of the dialysis membrane during the pancreatin–bile digestion showed that dialyzed hydroxycinnamic acids, which are described as a bioavailable fraction, represented 42.28% and 10.17% of their initial content in FJ and PJ, respectively. This may suggest a higher contribution of the native forms in the biological activity of FJ, and the unidentified metabolites in the case of PJ. According to Barak et al., the bioavailability index of chlorogenic acid was estimated as 38.90–56.40% after digestion of *V. opulus* fruit methanolic and aqueous extract [19].

As cellular models, we chose cells that are strongly involved in the regulation of glucose and lipid homeostasis, such as hepatic HepG2 cells, myoblast L6 cells, insulin secreting β-cells MIN6, and adipocytes 3T3-L1. The presented study indicated that in HepG2, L6, and 3T3-L1 cells, FJ and PJ from *V. opulus* fruit decreased intracellular oxidative stress. The preparation treated with the digestion process revealed antioxidant potential lower than the undigested samples. Some authors also indicated that the hepatic cellular antioxidant activity of the extract decreased after digestion, which is correlated with decreased amounts of phenolic compounds [40]. Jiao et al. demonstrated that blueberry phenolic compounds after in-vitro-simulated gastrointestinal digestion still had antioxidant activity against HepG2 cells, but it was 75% lower. Furthermore, among the identified phenolic compounds in the samples after gastrointestinal digestion, the authors identified chlorogenic acid, neochlorogenic acid, and a number of quercetin derivatives [41]. These phenolic acids have been also identified in tested *V. opulus* juice samples. Other in vitro studies suggested that hydroxycinnamic acids may constitute the main group of antioxidants in plant-origin food. These compounds are absorbed in the alimentary tract, with significant antioxidant properties [42]. It is worth noting that in the digested *V. opulus* juice samples, the abundant phenolic group was still hydroxycinnamic acids. In the presented study, usually, the highest biological effect was observed for PJ. The observed dependencies were the result of a 80-fold increase in the content of phenolic compounds after the FJ purification process on the Sep-Pak C18 column to PJ, which was described in previous research [10,15,16]. The digestion process lowered FJ and PJ activity. Nevertheless, research on *V. opulus* juice samples after the in vitro digestion process confirmed the bioactivity and availability of PJ. These features had a positive impact on the inhibition of induced steatosis of HepG2 hepatic cells, which was accompanied by lowered intracellular oxidative stress and improved glucose uptake. It is worth noting that in the literature, there are not many reports indicating the cytoprotective effects of digested plant extracts on FFA-induced steatosis conditions, and this is the first in vitro study demonstrating the positive effects of digested *V. opulus* juice rich in hydroxycinnamic acids in metabolic dysregulation in HepG2 cells. Yao et al. showed that digested buckwheat rich in transported hydroxycinnamic acids in Caco-2/HepG2 coculture models had lipase inhibitory activity, and reduced the levels of triglycerides, low-density lipoprotein cholesterol, and total cholesterol [43]. This paper also confirmed the bioavailability and lipid-lowering effects of plant-originated phenolic compounds, especially hydroxycinnamic acids. Moreover, hydroxycinnamic acids were not only effective in the reduction of lipid accumulation but also upregulated the glucose transporter 4 (GLUT4) expression and glucose uptake [44], which is in agreement with observed enhanced 2-NBDG analogue uptake by HepG2 treated with PJ and DPJ.

Analyzing the phenolic composition of the digested *V. opulus* juice samples, it can be speculated that chlorogenic acid (5-*O*-caffeoylquinic acid—5-CQA) and its isomers—neochlorogenic (3-CQA) and cryptochlorogenic acids (4-CQA)—were responsible for the presented activities. Chlorogenic acid itself is relatively stable in the stomach at low pH in humans and is hydrolyzed in the lower sections of the alimentary tract to caffeic and quinic acids [45]. Nevertheless, chlorogenic acid can be absorbed intact from the small intestine and demonstrates biological activities [46]. It needs to be emphasized that 5-CQA is isomerized during in vitro digestion to 3-CQA and 4-CQA, as has been indicated by many reports [47,48,49] also for digested chokeberry juice rich in 5-CQA, 3-CQA, anthocyanins, and flavonols [50]. Furthermore, higher content of 5-CQA after the digestion process of chokeberry juice and lower 3-CQA content were indicated [50]. Our results demonstrated the opposite effect, but we can speculate about the isomerization of 5-CQA to 3-CQA with an accompanying higher content of 4-CQA. Similarly, Bouayed et al., in juice from apple, observed the degradation of chlorogenic acid in the comparatively alkaline pH of the small intestine with its isomerization to neochlorogenic and cryptochlorogenic acids [51]. Due to the fact that 5-CQA conversion to 3-CQA and 4-CQA was also identified in rat hepatocytes and in human serum after consumption of apple juice [49], we can speculate that the presented hepatic activities of DPJ resulted from the bioavailability of identified *V. opulus* caffeoylquinic acid derivatives.

Lipotoxicity, characterized by the accumulation of ectopic lipids in skeletal muscle, is a major factor in the etiologies of FFA-induced insulin resistance, type 2 diabetes, and other metabolic dysfunction in skeletal muscle [27]. There is an increasing demand for compounds, including drugs and functional foods, that can prevent myocellular insulin resistance. Additionally, the free fatty acid oversupply resulting from lipoprotein lipase in the liver, as well as enhanced FFA transport into cells under the condition of insulin resistance, consequently lead to reduced mitochondrial muscle FFA oxidation, excess lipid accumulation in the cytosol, enhanced oxidative stress, and mitochondrial-induced apoptosis [24,28]. In the presented study, it was confirmed that *V. opulus* phenolics, especially the bioavailable fraction of DPJ, protected L6 myoblast cells against lipid accumulation and damage caused by ROS elevation. Some reports indicated that phenolic compounds from plant extracts have strong antioxidant activity in skeletal muscle cells [52,53,54], with enhanced glucose uptake and lowered FFA-induced lipid accumulation [55]. However, this is the first study identifying *V. opulus* phenolic compounds as cytoprotective agents against lipotoxicity induced by FFA in skeletal muscle cells. Ho et al. showed that cyanidin-3-*O*-glucoside, cyanidin-3-*O*-sambubioside, procyanidin B2, procyanidin C1, and some hydroxycinnamic acid derivatives extracted from elderberry had strong antioxidant activity and the ability to decrease oleic acid uptake, and they significantly increased glucose uptake by human skeletal muscle cells [52]. This result is in agreement with the presented study for FJ and PJ, in which we identified the same types of phenolic compounds; therefore, we can speculate that they may be responsible for the observed activities in L6 cells. Chlorogenic acid, an abundant phenolic compound in *V. opulus* juice samples, after the in vitro digestion process, also improved muscle function by regulating mitochondrial function and cellular energy metabolism [56,57]. Ong et al. showed that, in mice, chlorogenic acid improved skeletal muscle glucose uptake, which in turn improved the fasting glucose level, glucose tolerance, insulin sensitivity, and dyslipidemia [58]. In addition, the same authors indicated that chlorogenic acid increased glucose transport in skeletal muscle via AMP-activated protein kinase (AMPK) activation, with enhanced levels of phosphorylated acetyl-CoA carboxylase (pACC) [59]. Additionally, other research indicates that many phenolic compounds have the ability to influence the level of proteins involved in lipid metabolism and the insulin receptor pathway in muscle cells. Chen et al. proved that berberine improved FFA-induced insulin resistance in L6 myotubes through the inhibition of peroxisome proliferator-activated receptor gamma (PPARγ, regulator of FFA uptake and promotor of adipogenesis) and fatty acid transferase (FAT/CD36) [55]. Deng et al. showed that polyphenols suppressed PA-induced insulin resistance in C2C12 mouse skeletal muscle cells by enhanced phosphorylation of insulin receptor and increased levels of pACC with activation of AMPK via phosphorylation [60]. It is worth mentioning that a previous study indicated *V. opulus* juice phenolic compounds as inhibitors of PPARγ in 3T3L1 cells [10]. A study on HepG2 also showed enhanced pAMPK and pACC levels with increased levels of p-IRS induced by *V. opulus* phenolic compounds [17]. These results complement each other and lead to a conclusion that the cytoprotective activity of *V. opulus* juice in L6 cells against FFA-induced steatosis may be the reason for some of the molecular mechanisms involving the AMPK pathway or other protein factors involved in lipid and glucose metabolism. To prove this hypothesis, additional studies on protein expression must be performed in future research.

The effect of the digestion process of *V. opulus* fruit juice samples on adipogenesis regulation in 3T3-L1 cells was also studied. As shown previously, *V. opulus* phenolic compounds were able to decrease the differentiation of mouse preadipocytes [9,10], which was followed by the reduction of the activity and protein level of PPARγ nuclear receptor regulating the expression of proteins involved in lipid metabolism. In the observed process, other molecular regulators of preadipocyte differentiation may be involved, such as C/EBP or SRBP-1c proteins. Nevertheless, the decrease in cellular lipid accumulation was followed by a reduction in ROS generation, which in turn downregulated the secretion of proinflammatory molecules IL-6 and TNF-α. The sustained anti-inflammatory activity of digested *V. opulus* juice samples (DFJ, DPJ) may be correlated with the presence of chlorogenic acid, which has been demonstrated as a reducer of the cellular release of TNF-α, IL-1, and IL-6 cytokine [61]. Increased levels of both factors, IL-6 and TNF-α, have been diagnosed in people with obesity and osteopenia [62]. The reduction of the secretion of IL-6 and TNF-α by *V. opulus* juice was observed in Saos-2 cells, therefore showing that the juice may be important not only for the metabolism of bone tissue but also may delay its demineralization resulting from obesity [63]. Despite the observed positive, or at least neutral, effects of the digestion process on *V. opulus* fruit juice’s biological activity, there was one negative aspect determined. Previously, it was shown that *V. opulus* juice might induce MIN6 cells’ functional failure through the increase of ROS generation, FFA uptake, and, finally, the reduction of GSIS [16]. These results suggested that *V. opulus* juice could be involved in insulin resistance development and hyperglycemia elevation. Here, it was demonstrated that the digestion process of *V. opulus* juice had no protective effect on MIN6 cells’ functionality in regard to the studied activities; therefore, pancreas damage may be caused by its elevated consumption.

## 4. Materials and Methods

### 4.1. Chemicals and Reagents

Reference phenolic compounds (chlorogenic acid, neochlorogenic acid, and cryptochlorogenic acid) were obtained from Phytolab (Vestenbergsgreuth, Germany). Pancreatin from porcine pancreas (P1625), α-amylase from porcine pancreas (A3176-1Mu), pepsin from porcine gastric mucosa (P6887), mucin from porcine stomach (M2378), and bile from bovine and ovine (B8381) were provided by Sigma-Aldrich (Steinheim, Germany). All other reagents used in our study, if not stated otherwise, were also obtained from Sigma-Aldrich. All cell culture reagents were obtained from Life Technologies (Carlsbad, CA, USA). Tissue culture plastics were supplied by Greiner Bio-One GmbH (Frickenhausen, Austria). All the experimental measurements, if not stated otherwise, were performed using the Synergy 2 BioTekMicroplate Reader (BioTek, Winooski, VT, USA).

### 4.2. Preparation of V. opulus Samples

Fresh *V. opulus L.* fruits were obtained from Rogów Arboretum, Warsaw University of Life Sciences (Rogów, Poland) (account number 18162). The procedure for obtaining *V. opulus* fruit juices used in this study is described elsewhere [10]. The fresh juice (FJ) was obtained as a result of centrifugation (5000 rpm for 10 min) of homogenized fresh fruit. It was determined that 1 mL of FJ preparation contained 100 mg of dry weight. The purification process of FJ was carried out by solid-phase extraction with C-18 Sep-Pak cartridge (10 g capacity, Waters Corp., Milford, MA, USA) pre-treated with the application of methanol and water. FJ phenolic compounds loaded on the columns were eluted with methanol and, after removing the organic solvent under vacuum, the dry residue was diluted in water and freeze-dried to afford purified juice (PJ) sample. For biological activity assays, PJ was dissolved in a phosphate-buffered saline (PBS)/dimethylsulfoxide (DMSO) (1:1 *v*/*v*) mixture at concentration 100 mg/mL.

### 4.3. Simulated Gastrointestinal Digestion In Vitro

The in-vitro-simulated digestion model consisting of a three-step procedure that simulated digestion in mouth, stomach, and small intestine was performed on FJ and aqueous PJ solution (1 mg/mL) as described previously [38,64] with some modifications. Briefly, 10 mL of FJ or 10 mL of PJ aqueous solution was adjusted to pH 6.8 (1 M NaHCO_3_) and mixed with 10 mg of mucin and 20 mg of α-amylase. The mixture was incubated for 5 min in a shaking water bath at 37 °C. For the gastric digestion step, the pH value of the mixture was adjusted to 2 with 6 M HCl and 2 mg of pepsin was added, and then it was incubated for 2 h in the shaking water bath at 37 °C. For the small intestinal procedure, after adjusting the pH of the gastric digesta to 7.5 (1 M NaHCO_3_), 4.5 mL of pancreatin and bile salts mixture (4 mg/mL pancreatin and 25 mg/mL bile salts) was added, and the mixture was placed in a dialysis tube (molecular mass cut-off 12 kDa). Next, this cellulose dialysis tube was placed in a 150 mL glass beaker filled with 25–30 mL of the phosphate buffer (pH 7.5) (volume equal to the volume of the digested mixture), and the beaker was sealed with parafilm. Sample was incubated for 2.5 h in the shaking water bath at 37 °C. The dialysis tube content was considered the part of digesta that reach the colon (IN fraction), whereas the OUT fraction (phosphate buffer fraction), which contained the compounds capable of crossing the membrane, was considered the bioavailable fraction of the *V. opulus* juices. To ensure the inactivation of the enzymes, both fractions were heated in 78 °C for 10 min [65]. After this, the entire volume of IN and OUT fractions was purified by solid-phase extraction using the Sep-Pak C18 cartridge (10 g capacity, Waters Corp., Milford, MA, USA) [39]. Phenolic compounds were bound to the C18 cartridge, while sugars and other polar compounds were removed with water (60 mL). Then, phenolic compounds were eluted with methanol (60 mL). After methanol removal under reduced pressure (T < 40 °C), solid residue was dissolved in 5 mL of water and used for further analysis. The bioavailable fractions, called digested fresh juice (DFJ) and purified digested juice (PDJ), were used in the biological study.

### 4.4. Identification and Quantification of Phenolic Compounds by UPLC–PDA-Q/TOF-MS

The *V. opulus* FJ and PJ before and after digestion process were analyzed for the composition of hydroxycinnamic acids using the Acquity ultraperformance liquid chromatography (UPLC) system coupled with a quadruple-time of flight mass spectrometry (Q/TOF-MS) instrument (Waters Corp., Milford, MA, USA) equipped with an electrospray ionization (ESI) source as described previously [10]. Separations of individual phenolic acids were carried out using an Acquity UPLCR HSS T3 C18 column (150 × 2.1 mm, 1.8 µm; Corp., Milford, MA, USA) at 30 °C. The mobile phase was a mixture of 0.1% formic acid (A) and acetonitrile (B). The gradient program was as follows: initial conditions 99% (A), 12 min 65% (A), 12.5 min 100% (B), 13.5 min 99% (A). The flow rate was 0.45 mL/min and the injection volume was 5 µL. The mass spectrometer was operating in the negative mode for a mass range of 150–1500 Da, fixed source temperature at 100 °C, desolvation temperature 250 °C, desolvation gas flow of 600 L/h, cone voltage of 45 V, capillary voltage of 2.0 kV, collision energy 50 V. Leucine enkephalin was used as a lock mass. The single phenolic acids were characterized based on the retention times and the accurate molecular masses. The data obtained from UPLC–MS were analyzed in the Mass-LynxTM V 4.1 software. Phenolic acids were monitored at 320 nm. The PDA spectra were measured over the wavelength range 200–600 nm. Calibration curves were run for the external standards: neochlorogenic acid, chlorogenic acid, and cryptochlorogenic acid.

### 4.5. Cell Culture and Exposure Conditions

Human hepatoma HepG2 cell line was obtained from Leibniz Institute DSMZ—German Collection of Microorganisms and Cell Cultures (Leibniz, Germany) and grown in RPMI 1640 medium supplemented with 10% fetal bovine serum (FBS) and antibiotics (100 U/mL penicillin, 100 µg/mL streptomycin, and 25 µg/mL amphotericin). Rat skeletal muscle myoblast L6 cell line was supplied by ECACC (Porton Down, UK) and grown in Dulbecco’s Modified Eagle’s Medium (DMEM) with high glucose level, 10% fetal bovine serum (FBS), and antibiotics. The murine-adherent insulinoma MIN6 cells were kindly provided by Dr Jun-ichi Miyazaki from the Division of Stem Cell Regulation Research, Osaka University, Japan [66] and grown in DMEM with high glucose level, 10% fetal bovine serum (FBS), antibiotics, and 50 µM β-mercaptoethanol. Mouse preadipocyte 3T3-L1 cell line was supplied by ATCC (Manassas, VA, USA). For adipocyte differentiation, a confluent culture of 3T3-L1 cells was grown for two days in a preadipocyte medium DMEM with 10% calf serum; then, the cells were stimulated with a differentiation medium with DMEM containing 10% fetal bovine serum (FBS), 1 µM dexamethasone, 0.5 mM methylisobutylxanthine (IBMX), and 1 µg/mL insulin for two days. After 48 h of incubation, the differentiation medium was replaced with DMEM containing 10% FBS and 1 µg/mL insulin. Cell medium was replaced every second day with the addition of compounds studied. Analyses were carried out 7 days after differentiation.

All cell culture experiments were performed in a humidified 5% CO_2_ and 95% atmosphere at 37 °C. If not stated otherwise, after 24 h of cell seeding, the medium was changed into serum-free medium and tested *V. opulus* juice samples were added for another 24 h. For stimulation of steatosis of HepG2 and L6 cells, free fatty acids (oleic and palmitic acids—OA and PA) were added at concentration of 300 µM for HepG2 cells and at concentration of 100 µM OA and 75 µM PA for L6 cells. Tested fatty acids were dissolved in 100% methanol at concentration of 100 mM and were further diluted with culture medium. Tested *V. opulus* juice preparations were dissolved in 50% DMSO in PBS at concentration of 200 mg/mL and further diluted with culture medium. The highest percentage of methanol and DMSO did not exceed 0.005% and did not affect the metabolic activity of cells.

All the experimental measurements were performed using the Synergy 2 BioTek Microplate Reader (BioTek, Winooski, VT, USA). Microscopic observations were performed using fluorescent microscope Nikon TS100 Eclipse (Nikon, Tokyo, Japan) under 200× magnification. All cell culture reagents were obtained from Life Technologies (Carlsbad, CA, USA).

### 4.6. Cell Viability

Metabolic activity was evaluated with fluorescent measurements with PrestoBlue (Life Technologies, Carlsbad, CA, USA) according to the manufacturer’s instructions. Cells were seeded into 96-well plates at 1 × 10^4^ cells/well density in complete medium and grown overnight and then incubated in the presence of studied *V. opulus* juice samples for another 24 h, if not stated otherwise. After this, fluorescent reagent was added for 30 min and fluorescent signal at F530/590 nm was measured.

### 4.7. Detection of Intracellular Reactive Oxygen Species Generation

The effect of *V. opulus* juice samples on intracellular generation of reactive oxygen species (ROS) was investigated using dichloro-dihydro-fluorescein diacetate (DCFH-DA) chemical. Cells were seeded into a 96-well plate at a density of 1 × 10^4^ cells/well overnight. After 24 h, tested preparations were added and cells were incubated with samples for another 24 h. After the cells’ treatment with preparations, the cells were washed with phosphate-buffered saline (PBS) and incubated with 10 µM DCF for 30 min. For positive control, tert-BOOH (t-BOOH) was used at a concentration of 500 µM. Fluorescent signal at F485/530 nm was analyzed.

### 4.8. Determination of Lipid Accumulation and Fatty Acid Uptake

Cells were seeded into 96-well plate at a density of 2 × 10^4^ cells/well and grown to full confluence for each of experiment (2–3 days). After reaching confluence, cells were incubated in serum-free medium for 24 h with the *V. opulus* juice samples or/with the presence of tested fatty acid. After treatment, cells were washed with cold PBS, fixed in 5% paraformaldehyde for 30 min, and stained with Nile Red dye at the final concentration of 1 µg/mL for 40 min. The lipid-bound Nile Red fluorescent signal at F485/530 was measured. In regard to 3T3-L1 cells, the level of lipid accumulation was determined at the 7th day after adipogenesis induction. Fatty acid uptake was measured using Fatty Acid Uptake Kit (Sigma Aldrich, Steinheim, Germany). After the cells’ treatment with *V. opulus* juice preparations, a fluorescent probe TF2-C12 was added to serum-free medium and the fluorescent signal at F485/530 nm was measured after 1 h incubation with fluorescent analogue.

### 4.9. Glucose Uptake

Cells were seeded into 96-well plate at density of 1 × 10^4^ cells/well and incubated for 24 h. Briefly, after 24 h of treatment with *V. opulus* juice samples or/with tested fatty acids in serum-free medium, 150 µM of fluorescent glucose analogue 2-NBDG (2-(N-(7-nitrobenz-2-oxa-1,3-diazol-4-yl)amino)-2-deoxyglucose) was added in glucose- and serum-free medium. After 2 h of incubation with NBDG, cells were washed twice with serum- and glucose-free medium and fluorescent signal at F485/530 nm was measured immediately.

### 4.10. Insulin Secretion

Cells were seeded on 24-well plate at density 2 × 10^5^ cells/well and cultured 48 h before the experiment. Then, they were pre-incubated for 1 h with buffer (25 mM HEPES, 125 mM NaCl, 6 mM KCl, 1.2 mM MgCl_2_·6H_2_O, 1.3 mM CaCl_2_·2H_2_O, 2 mM glucose; pH 7.4). Subsequently, cells were incubated with *V. opulus* juice samples for 1 h, and buffer samples were collected. The same cells were incubated for another 1 h with fresh buffer containing 20 mM glucose tested preparations, and buffer samples were collected. The insulin secreted in buffers was measured with a Mercodia Mouse Insulin ELISA kit (Mercodia AB, Uppsala, Sweden) according to the manufacturer’s procedure. To normalize insulin level, in cell lysates obtained with 0.1% Triton X-100 with PBS, the protein content was quantified with a Bradford assay.

### 4.11. Statistical Analysis

Unless stated otherwise, all the biological results are presented as means of 3–6 repeated experiments ± SEM. All calculations were evaluated for significance using one-way ANOVA followed by Dunnett’s test with the GraphPad Prism 6.0 software (GraphPad Software, Inc., La Jolla, CA, USA). *p* ≤ 0.05 was considered statistically significant.

## 5. Conclusions

In this study, we examined the effect of in vitro mouth–gastric–intestine digestion on the biological activity of phenolic components present in *V. opulus* fresh juice and in a phenolic-rich fraction obtained from the juice. The results demonstrated that the juice matrix is an important factor influencing the stability of hydroxycinnamic acids, quantitatively the main phenolic component of the tested samples. Additionally, the *V. opulus* digested juices revealed biological potential in all studied cellular models. After cells’ treatment with the bioavailable juice fractions, the decrease in intracellular oxidative stress and the lipid accumulation were sustained, and an enhancement in glucose uptake was observed. The adipogenesis process was downregulated, which was followed by a reduction in the secretion of inflammatory cytokines. However, all studied juices, with or without digestion treatment, showed lipotoxic potential against pancreatic beta MIN6 cells, and they decreased the GSIS process. The observed in vitro biological activity clearly indicates the sustaining of the biological effectiveness of *Viburnum opulus* juice after its digestion process. However, due to *V. opulus*’ cytotoxic potential against pancreatic cells, its usage as a dietary supplement component in metabolic disorder prevention needs further evaluation. Future studies should also focus on the activity of the colon available compounds (IN fraction) after their treatment with gut microbiota.

## Figures and Tables

**Table 1:** Quantification of individual hydroxycinnamic acids (mg/g undigested sample) in *V. opulus* fresh juice (FJ) and purified juice (PJ) before and after in vitro digestion.

Peak	Compound	l_max_	[M − H]^−^ (*m*/*z*)	Sample		
				Undigested	OUT Fraction	IN Fraction
Fresh juice (FJ)						
1	Caffeoylquinic acid derivative I ^1^	337	353	n.d.	0.021 ± 0.008	n.d.
2	Neochlorogenic acid	323	353	0.007 ± 0.001	0.862 ± 0.067	0.582 ± 0.007
3	Caffeoylquinic acid derivative II ^1^	323	707	0.015 ± 0.000	n.d.	n.d.
4	Caffeoylquinic acid derivative III ^1^	323	707	0.024 ± 0.002	n.d.	n.d.
5	Caffeoylquinic acid derivative IV ^1^	325	707	0.017 ± 0.001	0.173 ± 0.010	0.122 ± 0.015
6	Chlorogenic acid	324	353/707	8.039 ± 0.147	1.222 ± 0.129	0.612 ± 0.018
7	Cryptochlorogenic acid	325	353	0.004 ± 0.000	0.687 ± 0.087	0.435 ± 0.009
8	Caffeoylquinic acid derivative V ^1^	325	705	n.d.	0.195 ± 0.059	0.091 ± 0.001
9	Caffeoylquinic acid derivative VI ^1^	319	705	n.d.	0.166 ± 0.048	0.064 ± 0.001
10	Caffeoylquinic acid	313	353	0.745 ± 0.001	0.126 ± 0.049	n.d.
11	NI	323	531	0.017 ± 0.001	0.348 ± 0.059	0.259 ± 0.011
12	Caffeoylquinic acid derivative VII ^1^	321	705	0.034 ± 0.000	n.d.	n.d.
13	Caffeoylquinic acid derivative VIII ^1^	320	705	0.034 ± 0.000	0.178 ± 0.038	0.165 ± 0.012
Purified juice (PJ)						
1	Caffeoylquinic acid derivative I ^1^	337	353	n.d.	0.859 ± 0.050	0.070 ± 0.001
2	Neochlorogenic acid	322	353	0.215 ± 0.019	19.883 ± 0.295	4.141 ± 0.014
3	Caffeoylquinic acid derivative II ^1^	323	707	1.289 ± 0.058	n.d.	n.d.
4	Caffeoylquinic acid derivative III ^1^	323	707	1.051 ± 0.008	n.d.	n.d.
5	Caffeoylquinic acid derivative IV ^1^	323	707	1.220 ± 0.020	1.367 ± 0.148	0.256 ± 0.002
6	Chlorogenic acid	318	353/707	645.492 ± 1.984	21.610 ± 0.040	3.330 ± 0.072
7	Cryptochlorogenic acid	323	353	0.484 ± 0.023	21.591 ± 0.072	5.902 ± 0.121
8	Caffeoylquinic acid ^1^	313	353/707	44.344 ± 0.176	2.019 ± 0.467	0.328 ± 0.088
9	Caffeoylquinic acid derivative V ^1^	325	705	3.306 ± 0.014	0.630 ± 0.065	0.324 ± 0.039
10	Caffeoylquinic acid derivative VI ^1^	325	705	3.268 ± 0.010	0.839 ± 0.067	0.766 ± 0.034
11	Feruloylquinic acid I ^1^	325	367	5.722 ± 0.021	1.387 ± 0.003	0.671 ± 0.064
12	Feruloylquinic acid II ^1^	304	367	0.528 ± 0.005	1.716 ± 0.266	n.d.

NI—not identified; n.d.—not detected; ^1^—equivalents of chlorogenic acid; results are expressed as a mean ± standard deviation (*n* = 3).

## Data Availability

Not applicable.

## Sample Availability

Samples of the compounds are not available from the authors.

## References

[1] Kajszczak D., Zakłos-Szyda M., Podsędek A. (2020). *Viburnum opulus* L.—A review of phytochemistry and biological effects. Nutrients.

[2] Rop O., Reznicek V., Valsikova M., Jurikova T., Mlcek J., Kramarova D. (2010). Antioxidant properties of European cranberrybush fruit (*Viburnum opulus* var. edule). Molecules.

[3] Perova I.B., Zhogova A.A., Cherkashin A.V., Éller K.I., Ramenskaya G. (2014). V Biologically active substances from European guelder berry fruits. Pharm. Chem. J..

[4] Baschali A., Tsakalidou E., Kyriacou A., Karavasiloglou N., Matalas A.L. (2017). Traditional low-alcoholic and non-alcoholic fermented beverages consumed in European countries: A neglected food group. Nutr. Res. Rev..

[5] Wójcik-Bojek U., Rywaniak J., Bernat P., Podsędek A., Kajszczak D., Sadowska B. (2021). An In vitro study of the effect of *Viburnum opulus* extracts on key processes in the development of Staphylococcal infections. Molecules.

[6] Sagdic O., Aksoy A., Ozkan G. (2006). Evaluation of the antibacterial and antioxidant potentials of cranberry (gilaburu, *Viburnum opulus* L.) fruit extract. Acta Aliment..

[7] Česonienė L., Daubaras R., Kraujalytė V., Venskutonis P.R., Šarkinas A. (2014). Antimicrobial activity of *Viburnum opulus* fruit juices and extracts. J. Verbrauch. Leb..

[8] Zakłos-Szyda M., Majewska I., Redzynia M., Koziołkiewicz M. (2015). Antidiabetic effect of polyphenolic extracts from selected edible plants as α-amylase, α-glucosidase and PTP1B inhibitors, and β pancreatic cells cytoprotective agents-a comparative study. Curr. Top. Med. Chem..

[9] Podsędek A., Zakłos-Szyda M., Polka D., Sosnowska D. (2020). Effects of *Viburnum opulus* fruit extracts on adipogenesis of 3T3-L1 cells and lipase activity. J. Funct. Foods.

[10] Zakłos-Szyda M., Pietrzyk N., Szustak M., Podsędek A. (2020). *Viburnum opulus* L. juice phenolics inhibit mouse 3T3-L1 cells adipogenesis and pancreatic lipase activity. Nutrients.

[11] Polka D., Podsedek A. (2019). Phenolics composition and antioxidant capacity of guelder rose fruit, flower and bark extracts. Biotechnol. Food Sci..

[12] Soylak A., Elci L., Saracoglu S., Divrikli U. (2002). Chemical analysis of fruit juice of European cranberrybush (*Viburnum opulus*) from Kayseri-Turkey. Asian J. Chem..

[13] Lachowicz S., Oszmianski J. (2018). The influence of addition of cranberrybush juice to pear juice on chemical composition and antioxidant properties. J. Food Sci. Technol..

[14] Çemtekİn B., Kilinç E., Karabacak L., Dağtekİn T. (2019). Aa evaluationof guelder rose (*Viburnum opulus* L.) and hawthorn (*Crataegus monogyna*) concentrates as alternative antioxidant sources to BHT and nitrite in poultry meat model system. Sci. Pap. Ser. D Anim. Sci..

[15] Zakłos-Szyda M., Pawlik N., Polka D., Nowak A., Koziołkiewicz M., Podsędek A. (2019). *Viburnum opulus* fruit phenolic compounds as cytoprotective agents able to decrease free fatty acids and glucose uptake by Caco-2 cells. Antioxidants.

[16] Zakłos-Szyda M., Kowalska-Baron A., Pietrzyk N., Drzazga A. (2020). Evaluation of *Viburnum opulus* L. fruit phenolics cytoprotective potential on insulinoma MIN6 cells relevant for diabetes mellitus and obesity. Antioxidants.

[17] Pietrzyk N., Zakłos-Szyda M., Koziołkiewicz M., Podsędek A. (2021). *Viburnum opulus* L. fruit phenolic compounds protect against FFA-induced steatosis of HepG2 cells via AMPK pathway. J. Funct. Foods.

[18] Karakurt S., Abuşoğlu G., Arituluk Z.C. (2020). Comparison of anticarcinogenic properties of *Viburnum opulus* and its active compound p-coumaric acid on human colorectal carcinoma. Turkish J. Biol..

[19] Barak T.H., Celep E., Yesilada E. (2019). Influence of in vitro human digestion on the bioavailability of phenolic content and antioxidant activity of *Viburnum opulus* L. (European cranberry) fruit extracts. Ind. Crop. Prod..

[20] Stanisavljević N., Samardžić J., Janković T., Šavikin K., Mojsin M., Topalović V., Stevanović M. (2015). Antioxidant and antiproliferative activity of chokeberry juice phenolics during in vitro simulated digestion in the presence of food matrix. Food Chem..

[21] Ombra M.N., Fratianni F., Granese T., Cardinale F., Cozzolino A., Nazzaro F. (2015). In vitro antioxidant, antimicrobial and anti-proliferative activities of purple potato extracts (*Solanum tuberosum* cv Vitelotte noire) following simulated gastro-intestinal digestion. Nat. Prod. Res..

[22] Gutiérrez-Grijalva E.P., Antunes-Ricardo M., Acosta-Estrada B.A., Gutiérrez-Uribe J.A., Basilio Heredia J. (2019). Cellular antioxidant activity and in vitro inhibition of α-glucosidase, α-amylase and pancreatic lipase of oregano polyphenols under simulated gastrointestinal digestion. Food Res. Int..

[23] Mantena S.K., King A.L., Andringa K.K., Eccleston H.B., Bailey S.M. (2008). Mitochondrial dysfunction and oxidative stress in the pathogenesis of alcohol- and obesity-induced fatty liver diseases. Free Radic. Biol. Med..

[24] Furukawa S., Fujita T., Shimabukuro M., Iwaki M., Yamada Y., Nakajima Y., Nakayama O., Makishima M., Matsuda M., Shimomura I. (2004). Increased oxidative stress in obesity and its impact on metabolic syndrome. J. Clin. Investig..

[25] Reynoso R., Salgado L.M., Calderón V. (2003). High levels of palmitic acid lead to insulin resistance due to changes in the level of phosphorylation of the insulin receptor and insulin receptor substrate-1. Mol. Cell. Biochem..

[26] Cao J., Feng X.X., Yao L., Ning B., Yang Z.X., Fang D.L., Shen W. (2014). Saturated free fatty acid sodium palmitate-induced lipoapoptosis by targeting glycogen synthase kinase-3β activation in human liver cells. Dig. Dis. Sci..

[27] Tumova J., Andel M., Trnka J. (2016). Excess of free fatty acids as a cause of metabolic dysfunction in skeletal muscle. Physiol. Res..

[28] Rachek L.I. (2014). Free fatty acids and skeletal muscle insulin resistance; *Prog*. Mol. Biol. Transl. Sci..

[29] Sawada K., Kawabata K., Yamashita T., Kawasaki K., Yamamoto N., Ashida H. (2012). Ameliorative effects of polyunsaturated fatty acids against palmitic acid-induced insulin resistance in L6 skeletal muscle cells. Lipids Health Dis..

[30] Wan L., Jiang J.G. (2018). Protective effects of plant-derived flavonoids on hepatic injury. J. Funct. Foods.

[31] Gião M.S., Pestana D., Faria A., Guimarães J.T., Pintado M.E., Calhau C., Azevedo I., Malcata F.X. (2010). Effects of extracts of selected medicinal plants upon hepatic oxidative stress. J. Med. Food.

[32] Villalpando-Arteaga E.V., Mendieta-Condado E., Esquivel-Solís H., Canales-Aguirre A.A., Gálvez-Gastélum F.J., Mateos-Díaz J.C., Rodríguez-González J.A., Márquez-Aguirre A.L. (2013). *Hibiscus sabdariffa* L. aqueous extract attenuates hepatic steatosis through down-regulation of PPAR-γ and SREBP-1c in diet-induced obese mice. Food Funct..

[33] Sousa J.N., Paraíso A.F., Andrade J.M.O., Lelis D.F., Santos E.M., Lima J.P., Monteiro-Junior R.S., D’Angelo M.F.S.V., de Paula A.M.B., Guimarães A.L.S. (2020). Oral gallic acid improve liver steatosis and metabolism modulating hepatic lipogenic markers in obese mice. Exp. Gerontol..

[34] Żyżelewicz D., Zakłos-Szyda M., Juśkiewicz J., Bojczuk M., Oracz J., Budryn G., Miśkiewicz K., Krysiak W., Zduńczyk Z., Jurgoński A. (2016). Cocoa bean (*Theobroma cacao* L.) phenolic extracts as PTP1B inhibitors, hepatic HepG2 and pancreatic β-TC3 cell cytoprotective agents and their influence on oxidative stress in rats. Food Res. Int..

[35] Chen Z., Yang Y., Mi S., Fan Q., Sun X., Deng B., Wu G., Li Y., Zhou Q., Ruan Z. (2019). Hepatoprotective effect of chlorogenic acid against chronic liver injury in inflammatory rats. J. Funct. Foods.

[36] Inada K.O.P., Silva T.B.R., Lobo L.A., Domingues R.M.C.P., Perrone D., Monteiro M. (2020). Bioaccessibility of phenolic compounds of jaboticaba (*Plinia jaboticaba*) peel and seed after simulated gastrointestinal digestion and gut microbiota fermentation. J. Funct. Foods.

[37] Correa-Betanzo J., Allen-Vercoe E., McDonald J., Schroeter K., Corredig M., Paliyath G. (2014). Stability and biological activity of wild blueberry (*Vaccinium angustifolium*) polyphenols during simulated in vitro gastrointestinal digestion. Food Chem..

[38] Mosele J.I., Macià A., Romero M.P., Motilva M.J., Rubió L. (2015). Application of in vitro gastrointestinal digestion and colonic fermentation models to pomegranate products (juice, pulp and peel extract) to study the stability and catabolism of phenolic compounds. J. Funct. Foods.

[39] Podsędek A., Majewska I., Redzynia M., Sosnowska D., Koziołkiewicz M. (2014). In vitro inhibitory effect on digestive enzymes and antioxidant potential of commonly consumed fruits. J. Agric. Food Chem..

[40] Boaventura B.C.B., Amboni R.D. (2015). de M.C.; da Silva, E.L.; Prudencio, E.S.; Di Pietro, P.F.; Malta, L.G.; Polinati, R.M.; Liu, R.H. Effect of in vitro digestion of yerba mate (*Ilex paraguariensis* A. St. Hil.) extract on the cellular antioxidant activity, antiproliferative activity and cytotoxicity toward HepG2 cells. Food Res. Int..

[41] Jiao X., Li B., Zhang Q., Gao N., Zhang X., Meng X. (2018). Effect of in vitro-simulated gastrointestinal digestion on the stability and antioxidant activity of blueberry polyphenols and their cellular antioxidant activity towards HepG2 cells. Int. J. Food Sci. Technol..

[42] Tagliazucchi D., Verzelloni E., Bertolini D., Conte A. (2010). In vitro bio-accessibility and antioxidant activity of grape polyphenols. Food Chem..

[43] Yao Y., Xu F., Ju X., Li Z., Wang L. (2020). Lipid-lowering effects and intestinal transport of polyphenol extract from digested buckwheat in Caco-2/HepG2 coculture models. J. Agric. Food Chem..

[44] Varshney R., Mishra R., Das N., Sircar D., Roy P. (2019). A comparative analysis of various flavonoids in the regulation of obesity and diabetes: An in vitro and in vivo study. J. Funct. Foods.

[45] Gumienna M., Lasik M., Czarnecki Z. (2011). Bioconversion of grape and chokeberry wine polyphenols during simulated gastrointestinal in vitro digestion. Int. J. Food Sci. Nutr..

[46] Olthof M.R., Hollman P.C.H., Katan M.B. (2001). Chlorogenic acid and caffeic acid are absorbed in humans. J. Nutr..

[47] Farah A., Guigon F., Trugo L.C. 5-Caffeoylquinic acid digestibility in human digestive fluids. Proceedings of the 21st International Conference on Coffee Science Colloquium.

[48] Stalmach A., Mullen W., Barron D., Uchida K., Yokota T., Cavin C., Steiling H., Williamson G., Crozier A. (2009). Metabolite profiling of hydroxycinnamate derivatives in plasma and urine after the ingestion of coffee by humans: Identification of biomarkers of coffee consumption. Drug Metab. Dispos..

[49] Kahle K., Kempf M., Schreier P., Scheppach W., Schrenk D., Kautenburger T., Hecker D., Huemmer W., Ackermann M., Richling E. (2011). Intestinal transit and systemic metabolism of apple polyphenols. Eur. J. Nutr..

[50] Bermúdez-Soto M.-J., Tomás-Barberán F.-A., García-Conesa M.-T. (2007). Stability of polyphenols in chokeberry (*Aronia melanocarpa*) subjected to in vitro gastric and pancreatic digestion. Food Chem..

[51] Bouayed J., Deußer H., Hoffmann L., Bohn T. (2012). Bioaccessible and dialysable polyphenols in selected apple varieties following in vitro digestion vs. their native patterns. Food Chem..

[52] Ho G.T.T., Kase E.T., Wangensteen H., Barsett H. (2017). Phenolic Elderberry extracts, anthocyanins, procyanidins, and metabolites influence glucose and fatty acid uptake in human skeletal muscle cells. J. Agric. Food Chem..

[53] Posadino A.M., Cossu A., Giordo R., Piscopo A., Abdel-Rahman W.M., Piga A., Pintus G. (2021). Antioxidant properties of olive mill wastewater polyphenolic extracts on human endothelial and vascular smooth muscle cells. Foods.

[54] Goutzourelas N., Stagos D., Spanidis Y., Liosi M., Apostolou A., Priftis A., Haroutounian S., Spandidos D.A., Tsatsakis A.M., Kouretas D. (2015). Polyphenolic composition of grape stem extracts affects antioxidant activity in endothelial and muscle cells. Mol. Med. Rep..

[55] Chen Y., Li Y., Wang Y., Wen Y., Sun C. (2009). Berberine improves free-fatty-acid-induced insulin resistance in L6 myotubes through inhibiting peroxisome proliferator-activated receptor γ and fatty acid transferase expressions. Metabolism..

[56] Ommati M.M., Farshad O., Mousavi K., Khalili M., Jamshidzadeh A., Heidari R. (2020). Chlorogenic acid supplementation improves skeletal muscle mitochondrial function in a rat model of resistance training. Biologia.

[57] Yun N., Kang J.W., Lee S.M. (2012). Protective effects of chlorogenic acid against ischemia/reperfusion injury in rat liver: Molecular evidence of its antioxidant and anti-inflammatory properties. J. Nutr. Biochem..

[58] Ong K.W., Hsu A., Tan B.K.H. (2013). Anti-diabetic and anti-lipidemic effects of chlorogenic acid are mediated by AMPK activation. Biochem. Pharmacol..

[59] Ong K.W., Hsu A., Tan B.K.H. (2012). Chlorogenic acid stimulates glucose transport in skeletal muscle via AMPK activation: A contributor to the beneficial effects of coffee on diabetes. PLoS ONE.

[60] Deng Y.T., Chang T.W., Lee M.S., Lin J.K. (2012). Suppression of free fatty acid-induced insulin resistance by phytopolyphenols in C2C12 mouse skeletal muscle cells. J. Agric. Food Chem..

[61] Gao R., Yang H., Jing S., Liu B., Wei M., He P., Zhang N. (2018). Protective effect of chlorogenic acid on lipopolysaccharide-induced inflammatory response in dairy mammary epithelial cells. Microb. Pathog..

[62] Shapses Sue A., Claudia P. (2017). ; Wang Yang Obesity is a concern for bone health with aging. Nutr Res..

[63] Zakłos-Szyda M., Nowak A., Pietrzyk N., Podsędek A. (2020). *Viburnum opulus* L. juice phenolic compounds influence osteogenic differentiation in human osteosarcoma Saos-2 cells. Int. J. Mol. Sci..

[64] Flores F.P., Singh R.K., Kerr W.L., Pegg R.B., Kong F. (2014). Total phenolics content and antioxidant capacities of microencapsulated blueberry anthocyanins during in vitro digestion. Food Chem..

[65] Yang I.F., Jayaprakasha G.K., Patil B.S. (2017). In vitro bile acid binding capacities of red leaf lettuce and cruciferous vegetables. J. Agric. Food Chem..

[66] Miyazaki J.I., Araki K., Yamato E., Ikegami H., Asano T., Shibasaki Y., Oka Y., Yamamura K.I., Miyazaki J.I. (1990). Establishment of a pancreatic β cell line that retains glucose-inducible insulin secretion: Special reference to expression of glucose transporter isoforms. Endocrinology.

